# The association between BMI and gallbladder cancer risk: a meta-analysis

**DOI:** 10.18632/oncotarget.9664

**Published:** 2016-05-27

**Authors:** Zhan-Ming Li, Zhao-Xia Wu, Bing Han, Yu-Qin Mao, Hui-Ling Chen, San-Feng Han, Jing-Lin Xia, Li-Shun Wang

**Affiliations:** ^1^ Institute of Biomedical Sciences, Minhang Hospital, Fudan University, Shanghai, P.R. China; ^2^ Ruijin Hospital, Shanghai Jiao-Tong University School of Medicine, Shanghai, P.R. China

**Keywords:** BMI, gallbladder cancer, meta-analysis, overweight, obese

## Abstract

Obesity is a known cause of gallstone formation and gallstones increases the risk of gallbladder cancer (GBC), but the relation of body mass index (BMI) to GBC remains incompletely understood. To help elucidate the role of obesity in GBC, we performed a meta-analysis of the relationship between BMI and GBC risk. PUBMED and EMBASE databases were searched up to April 17, 2016. Fifteen articles with 5902 cases were identified. Random-effects models and dose-response meta-analyses were used to pool study results. Compared to normal weight, the pooled relative risks (RRs) and the corresponding 95% confidence intervals (CI) of GBC for overweight and obesity is 1.10 (0.98-1.23) and 1.58 (1.43-1.75) respectively. The RRs and 95% CI of overweight and obesity in man are 0.98 (0.90-1.08) and 1.43 (1.19-1.71), while the corresponding RRs in woman are 1.29 (1.08-1.55) and 1.68 (1.41-2.00) when compared to normal weight. A nonlinear dose-response relationship between BMI and risk of GBC was found (P=0.001), and the risk increased by 4% for each 1 kg/m^2^ increment in BMI. When adjusted for sex, at the point of BMI=25 kg/m^2^, the RRs (95% CIs) for women and men were 1.13 (1.01-1.25) and 0.98 (0.90-1.07) respectively. The corresponding RRs (95%CIs) at the point of BMI=30 kg/m^2^ were 1.56(1.39-1.75) vs. 1.24(1.06-1.44). These results suggest that association of obesity and risk of GBC is stronger in woman. Furthermore, overweight is only associated with GBC in woman. A even stricter weight control might be necessary for woman to prevent GBC.

## INTRODUCTION

Gallbladder cancer (GBC) is the most common malignancy of the biliary tract, representing 80-95% of biliary tract cancers worldwide [1, 2]. This tumor ranks fifth among gastrointestinal cancers and it is traditionally regarded as a highly lethal disease with an overall 5-year survival of less than 5% [3]. The overall mean survival rate for patients with GBC is 6 months [4]. However, the causes for carcinogenesis of GBC are largely uncertain except the gallbladder stone [5].

The global prevalence of excess bodyweight in adults increased by 27.5% between 1980 and 2013, although the increase has slowed in recent years in some European countries and the USA [6-9], according to recent estimates [10, 11]. This problem is of great concern for public health, as excess body weight is known to be a major risk factor for cardiovascular disease, type 2 diabetes and certain cancer types [12, 13]. Overweight and obesity may also have an increased risk of developing GBC [14-16], but the relationship of body mass index (BMI) to GBC remains incompletely understood. For example, the sex difference for the risk of BMI on GBC had not been well elucidated. We therefore conduct a systematic meta-analysis to assess the associations of overweight and obesity with risk of GBC.

## RESULTS

### Literature search and study characteristics

**Figure 1:**
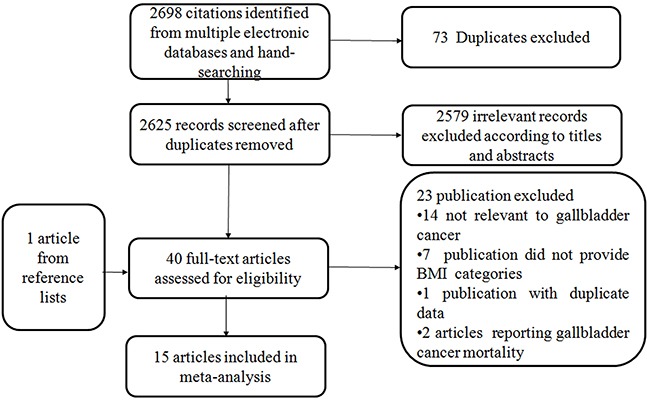
Flowchart of the selection of studies for inclusion in this meta-analysis

A total of 10 cohort studies [17-26] and 5 case-control studies [27-31] included in the meta-analysis and involved a total of 5902 cases (Figure 1). The duration of follow-up ranged from 4.8 to 23 years. Among these studies, 8 [17, 19, 21, 22, 27–30] in white, 5 [18, 23–26] in Asian, and 2 [20, 31] in mixed population. 3 studies [19, 20, 25] and 2 studies [26, 27] reported sole outcomes of males or females, respectively. 10 studies [17, 18, 21–24, 28–31] reported outcomes of both sex. Of the 10 studies, 7 [17, 18, 21–24, 31] reported outcomes of males and females separately while 3 studies [28-30] provided data of males and females combined. Main characteristics of the studies are shown in Table 1.

**Table 1:** Characteristics of the 15 included articles on BMI and risk of GBC

Author, year, country	Age ranges	Duration of follow-up	Study size no.	No of cases	Assessment method of weight/height	BMI (kg/m^2^)	RR(95%CIs)		Adjustment factors	NOS
							Men	Women		
Cohort studies										
Engeland et al, 2005 (Norway)	20–74, range	23	M: 963619 W:1037892	M: 628 W:1087	Measured	18.5-24.9 25.0-29.0 ≥30.0	1.00(Reference) 1.00 (0.84 – 1.17) 1.38 (1.01 – 1.89)	1.00(Reference) 1.27(1.10–1.47) 1.88 (1.60 – 2.21)	Age, birth cohort	7
Ishiguro et al, 2008 (Japan)	40-69, range	11.78	M: 48681 W: 53187	M:30 W: 63	Self-report	≤22.9 23.0-24.9 25.0-26.9 ≥27.0	1.00(Reference) 0.74(0.28-1.92) 1.26(0.48-3.33) 1.39 (0.45-4.34)	1.00(Reference) 0.47(0.22-0.98) 0.62(0.29-1.34) 0.94(0.48-1.88)	Age, gender, study area, cholelithiasis, smoking, alcohol	6
Jee et al, 2008 (Korea)	45.0 M 49.4 W , average	10.8	M: 770556 W: 443273	M: 2276 W:1062	Measured	<20.0 20.0–22.9 23.0–24.9 25.0–29.9 ≥30	0.8(0.68-0.94) 0.86(0.77-0.96) 1.00(Reference) 0.97(0.86-1.1) 1.65(1.11-2.44)	0.97(0.78-1.21) 1.12(0.9-1.41) 1.00(Reference) 1.27(1.02-2.12) 1.44(0.98-2.12)	Age, smoking	8
Kuriyama et al, 2005 (Japan)	40-70, range	9	M: 12485 W: 15054	M: 9 W: 24	Self-report	18.5-24.9 25.0-27.4 27.5-29.9 ≥30.0	1.00(Reference) 0.46(0.05–3.93)	1.00(Reference) 0.83(0.23-2.98) 3.43(1.19-9.94) 4.45 (1.39 −14.2)	Age, smoking, type of health insurance, intakes of alcohol, meat, fish, fruits, vegetables, bean paste soup	7
Moller et al, 1994 (Denmark)	50 M 60 W, average	4.8	M: 14531 W: 29434	M: 2 W: 26	Discharge diagnosis	Non-obese Obese	1.00(Reference) 0.5(0.1-1.8)	1.00(Reference) 1.4(0.9-2.1)	Age	6
Oh et al, 2005 (Korea)	≥20, range	10	M: 781283	M:182	Measured	18.5-22.9 23.0-24.9 25.0-26.9 ≥27.0	1.00(Reference) 1.55(1.1-2.2) 1.15(0.74-1.80) 1.25(0.70-2.24)	NA	Age, area of residence, smoking, exercise, alcohol	7
Samanic et al, 2004 (United States)	52.18 whites 47.63 blacks, average	12	M:3668486 M: 832214	M: 291 M: 47	Discharge diagnosis	White M Non-obese Obese Black M Non-obese Obese	White M 1.00(Reference) 1.70(1.13-2.57) Black M 1.00(Reference) 0.93(0.23-3.86)	NA	Age, calendar year	6
Samanic et al, 2006 (Sweden)	34.3, average	19	M: 362552	M: 109	Measured	18.5-24.9 25.0-29.0 >30.0	1.00(Reference) 0.93(0.62-1.39) 1.40(0.73-2.70)	NA	Age, smoking	8
Wolk et al, 2001 (Sweden)	24-51, range	10.3	M: 8165 W: 19964	M: 2 W: 29	Discharge diagnosis	Non-obese Obese	1.00(Reference) 0.9(0.1-3.4)	1.00(Reference) 1.7(1.1-2.5)	Age, calendar year	7
Song et al, 2008 Korea	40-64, range	8.75	W:170481	W: 88	Measured	<18.5 18.5-20.9 21.0-22.9 23.0-24.9 25.0-26.9 27.0-29.9 ≥30.0	NA	2.14(0.71-6.49) 1.28(0.59-2.78) 1.00(Reference) 1.03(0.52-2.03) 1.27(0.65-2.51) 1.59(0.79-3.22) 1.51(0.5-4.54)	Age, height, smoking status, alcohol intake, physical exercise, pay level at study entry	7
Case-control studies										
Grainge et al, 2009 (UK)	72, average		M and W: 3007	M and W: 241	Discharge diagnosis	<24.9 25.0-29.9 ≥30.0	M+W 1.00(Reference) 1.03(0.62-1.72) 1.51(0.83-1.75)	NA	Cigarette smoking, alcohol consumption	8
Nakadaira et al, 2009 (Hungry)	40-69, range		W: 37	W:41	Self-report	<24.9 25.0-29.9 ≥30.0	NA	1.00(Reference) 1.5(0.4-5.00) 0.8(0.3-1.80)	Age	7
Strom et al, 1995 (Mexico)	45-75, range		M and W: 110	M and W: 65	Self-report	<24.0 24.0-25.9 26.0-28.0 >28.0	M+W 1.00(Reference) 1.5(0.5-4.6) 2.2(0.7-8.4) 1.6(0.4-6.1)	NA	Age, sex, country	6
Serra et al, 2002 (Chile)	65.8 M 70.6 W, average		M and W:114	M and W: 114	Self-report	<25.0 25.0-29.9 ≥30.0	M+W 1.00(Reference) 0.8(0.4-1.4) 0.9(0.4-1.8)	NA	Age, sex	7
Zatonski et al, 1997 (Multicenter)	62.7 M 64.2 W, average		M: 798 W: 681	M: 44 W: 145	Self-report	Quartile 1 Quartile 2 Quartile 3 Quartile 4	1.0(reference) 1.0(0.3-3.0) 0.7(0.3-2.0) 1.0(0.3-2.8)	1.0(reference) 1.7(0.9-3.1) 1.5(0.8-3.0) 2.1(1.2-3.8)	Age, center, alcohol, smoking, schooling, and response status	7

BMI, body mass index; GBC, gallbladder cancer; RR, relative risk; CI, confidence interval; NA, not available; M, men; W, women; NOS, Newcastle-Ottawa Scale.

### Abnormal BMI and risk of GBC

**Figure 2:**
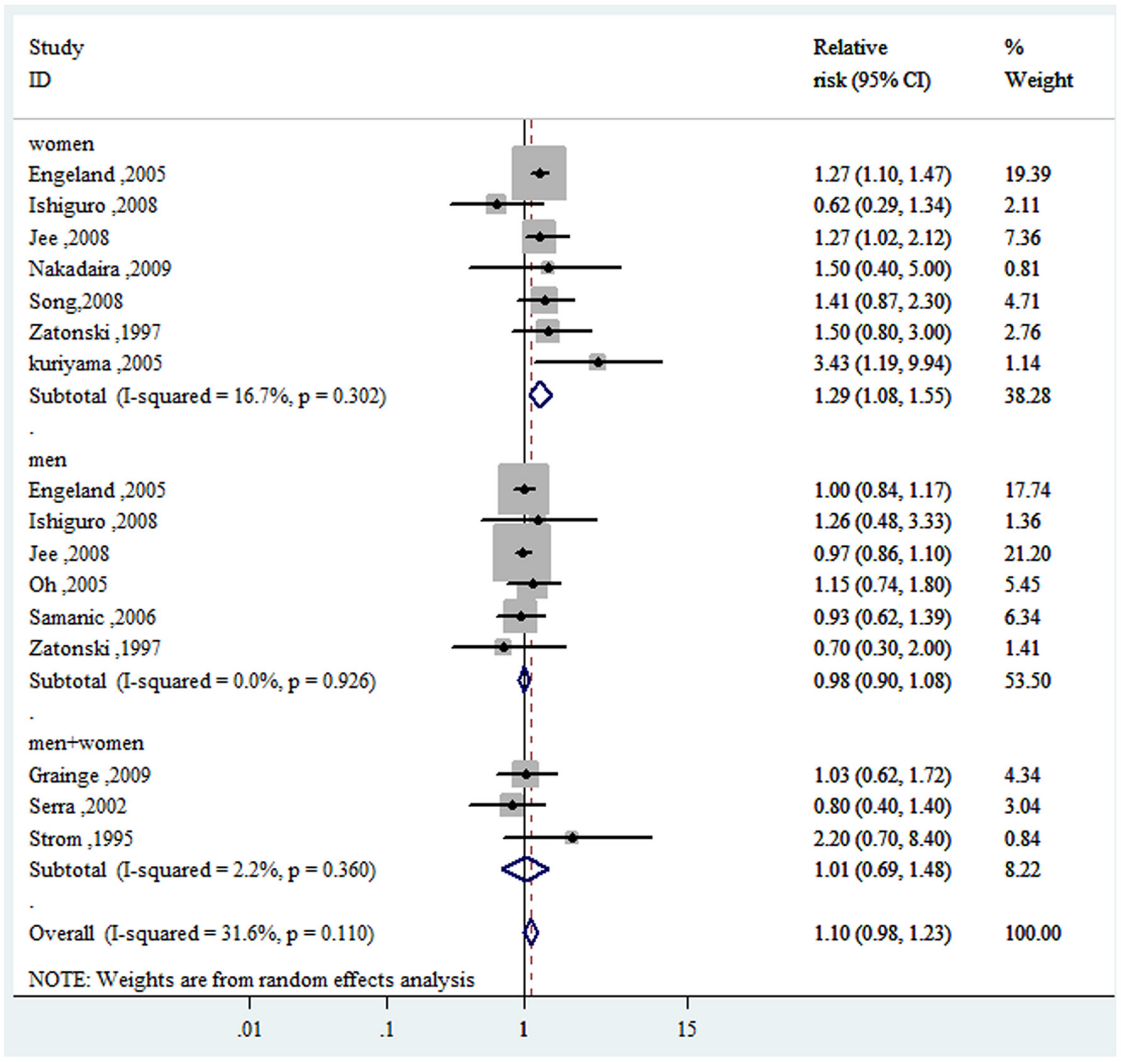
Forest plot of RRs of overweight *VS.* normal weight for BMI with GBC risk RR, relative risk; CI, confidence interval; BMI: body mass index; GBC, gallbladder cancer.

**Figure 3:**
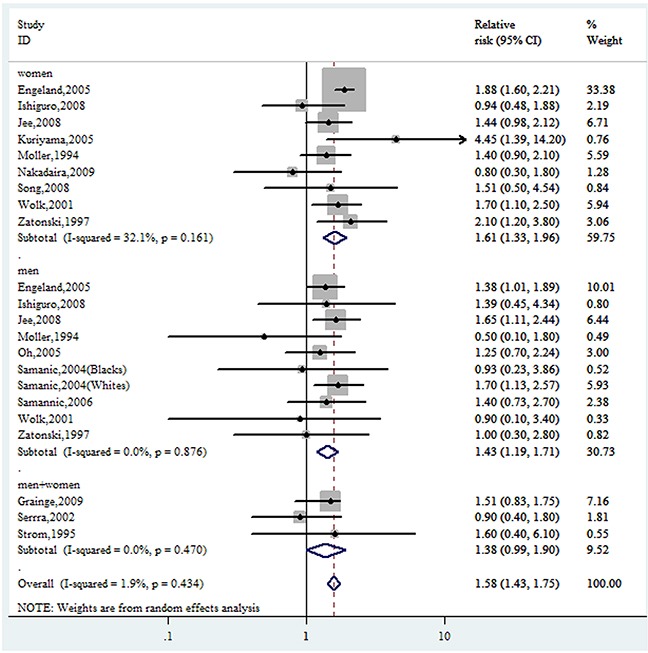
Forest plot of RRs of obesity *VS.* normal weight for BMI with GBC risk RR, relative risk; CI, confidence interval; BMI: body mass index; GBC, gallbladder cancer.

Compared to the reference category (normal weight), the combined RRs (95% CIs) of GBC were 1.10(0.98-1.23) and 1.58(1.43-1.75) for the category of overweight and obesity, respectively (Figure 2 & Figure 3). No evidence for high heterogeneity among studies was found in the analyses (overweight: I^2^=31.6%; obesity: I^2^= 1.9%).

### Subgroup analysis

For the category of overweight and obesity, subgroup analysis showed a basically consistent result with the overall analysis (Table 2). The risk of GBC with overweight and obesity was higher in women, in studies which adjusted for smoking, in studies which NOS quality score≥7 and in the obese population followed up over 12 years and in studies which located in Europe. Higher risk of GBC was also observed in cohort studies.

**Table 2:** Subgroup analyses of BMI and GBC

Study	Overweight			obesity		
	No. of studies	RR(95%CI)	I^2^	No. of studies	RR(95%CI)	I^2^(%)
All studies	12	1.10(0.98-1.23)	31.6	15	1.58(1.43-1.75)	1.9
**Sex**						
Men	6	0.98(0.90-1.08)	0.0	9	1.43(1.19-1.71)	0.0
Women	7	1.29(1.08-1.55)	16.7	9	1.68(1.41-2.00)	32.1
Combined	3	1.01(0.69-1.48)	2.2	3	1.38(0.99-1.90)	0.0
**Study location**						
Asia	5	1.14(0.91-1.44)	44.5	5	1.47(1.18-1.83)	0.0
Europe	4	1.11(0.95-1.28)	31.0	6	1.55(1.31-1.83)	23.1
**Study design**						
Cohort	7	1.11(0.96-1.27)	49.3	10	1.62(1.46-1.80)	0.9
Case-control	5	1.08(0.80-1.46)	0.0	5	1.40(1.06-1.84)	5.1
**Duration of follow-up(cohort studies only)**						
≥12	2	1.09(0.90-1.33)	63.5	3	1.71(1.49-1.98)	4.1
<12	5	1.14(0.91-1.44)	44.5	7	1.46(1.23-1.74)	0.0
**NOS quality score**						
≥7	10	1.10(0.98-1.24)	35.3	11	1.60(1.42-1.80)	8.5
<7	2	1.06(0.52-2.16)	38.7	4	1.37(1.07-1.76)	0.0
**Assessment method of weight/height**						
Self-reported	6	1.17(0.79-1.72)	35.0	6	1.33(0.91-1.95)	31.0
Measured	5	1.10(0.97-1.24)	45.8	5	1.67(1.48-1.89)	0.0
Discharge diagnosis	1	1.03(0.62-1.72)		4	1.51(1.24-1.84)	0.0
**Adjustment factors smoking**						
Yes	7	1.11(0.94-1.31)	27.7	7	1.56(1.30-1.86)	0.0
No	5	1.09(0.90-1.32)	36.6	8	1.47(1.24-1.74)	23.2
**Alcohol consumption**						
Yes	6	1.18(0.91-1.53)	36.6	6	1.50(1.17-1.91)	5.1
No	6	1.08(0.95-1.22)	42.4	9	1.59(1.41-1.78)	5.0

BMI, body mass index; GBC, gallbladder cancer; RR, relative risk; CI, confidence interval.

It is noted that the risk of gallbladder cancer for women were significantly higher than men both in the category of overweight (1.29 (1.08-1.55)(I^2^=16.7) vs. 0.98 (0.90-1.08)(I^2^=0.0)) and obese (1.61 (1.33-1.96) (I^2^=32.1) vs. 1.43 (1.19-1.71) (I^2^=0.0)) when adjusted for sex. Effect differences weren't observed for different BMI assessment method.

For overweight, some evidence of heterogeneity was found in studies of which duration of follow-up was more than 12 years (I^2^=63.5). No significant heterogeneity in obesity was found.

### Dose-response meta-analysis

**Figure 4:**
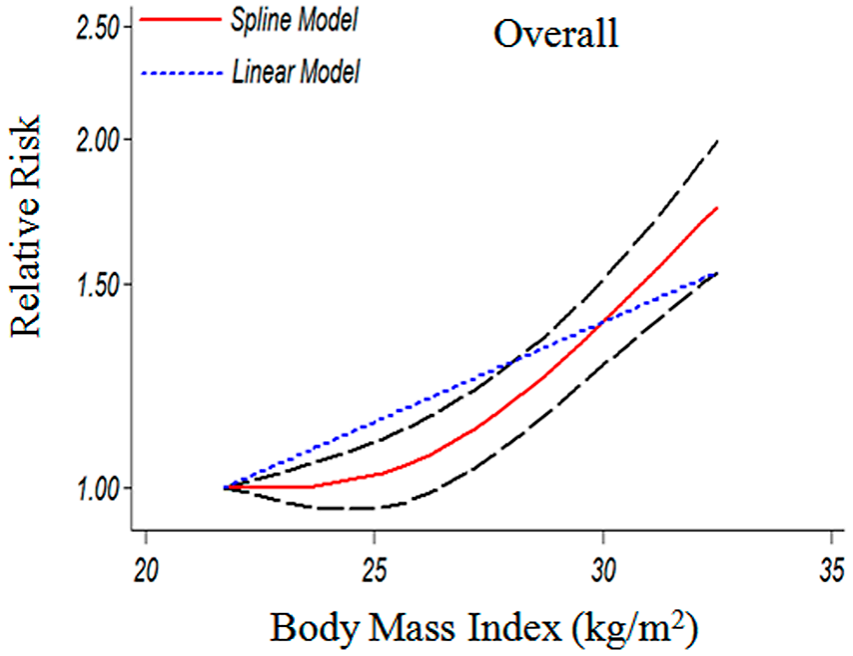
The dose-response analysis between BMI and GBC risk in cohort studies with restricted cubic splines in a multivariate random-effects dose-response model The solid line and the long dash line represent the estimated RR and its 95% CI (1.04(1.02-1.06) p=0.001). Short dash line represents the linear relationship (per 1 kg/m^2^ increment). RR, relative risk; CI, confidence interval; BMI: body mass index; GBC, gallbladder cancer.

**Figure 5:**
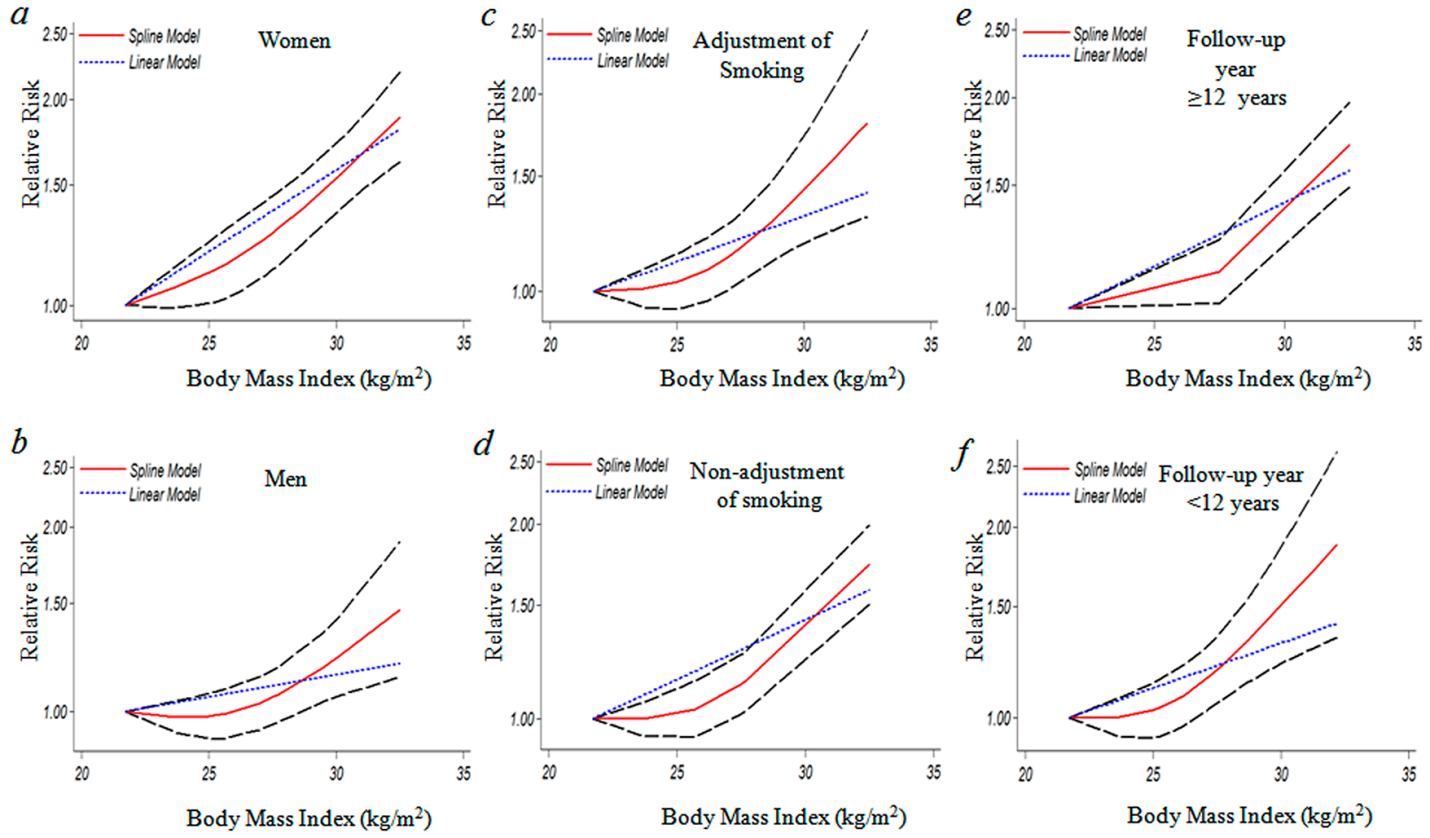
The dose-response analysis between BMI and GBC risk by adjustment of sex, smoking and duration of follow-up **a.** women (1.06(1.03-1.09) p=0.000); **b.** men (1.02(1.00-1.03) p=0.042); **c.** adjustment of smoking (1.04(1.01-1.07) p=0.006); **d.** non-adjustment of smoking (1.03(0.99-1.07) p=0.113); **e.** follow-up year ≥12 (1.03(0.99-1.07) p=0.065); **f.** follow-up year <12 years (1.04(1.01-1.07) p=0.011). The solid line and the long dash line represent the estimated RR and its 95% CI. Short dash line represents the linear relationship (per 1 kg/m^2^ increment). RR, relative risk; CI, confidence interval; BMI: body mass index; GBC, gallbladder cancer.

Seven studies [17–19, 23–26] were included in the dose-response meta-analysis of BMI and GBC. This meta-analysis showed an increased GBC risk of 1.04 (1.02-1.06) for each 1 kg/m^2^ increase in BMI as shown in Figure 4. When adjusted for sex, as shown in Figure 5a, the risk of GBC for each 1kg/m^2^ increase was more significantly increased in women than men (6% (RR=1.06, 95%CI=1.03-1.09) vs. 2% (RR=1.02, 95%CI=1.00-1.03). The dose-response associations were not affected by the adjustment of smoking and follow-up duration (Figure 5).

As shown in Figure 4, a significant nonlinear dose-response (P=0.001) relationship between BMI and risk of GBC was found. Compared to BMI=21.75 kg/m^2^, the summary RRs (95%CIs) of GBC were 1.03(0.96-1.10), 1.41(1.29-1.54) for BMI=25 and 30 kg/m^2^, respectively. A statistically more significant nonlinear relationship between BMI and GBC risk was observed in women when adjusted by sex (Figure 5). At the point of BMI=25 kg/m^2^, the RRs (95% CIs) for women and men were 1.13 (1.01-1.25) and 0.98 (0.90-1.07) respectively. The corresponding RRs (95%CIs) at the point of BMI=30 kg/m^2^ were 1.56(1.39-1.75) vs. 1.24(1.06-1.44) (Figure 5).

### Sensitivity analysis

In a sensitivity analysis in which one study at a time was removed and the rest analyzed, the pooled RRs ranged from 1.02 to 1.14 for overweight and from 1.45 to 1.61 for obesity, respectively, which indicated that the pooled estimates were stable and not influenced by a single study.

### Publication bias

**Figure 6:**
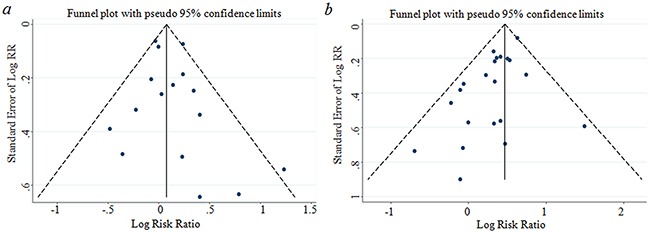
Funnel plot corresponding to the random-effects meta-analysis of the relationship between (a) overweight and GBC risk (p=0.398 by Egger's test); (b) obese and GBC risk(p=0.008 by Egger's test) GBC, gallbladder cancer.

**Figure 7:**
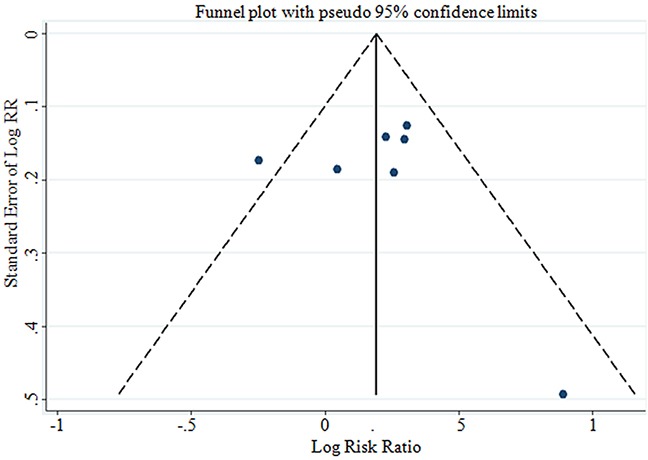
Funnel plot corresponding to the dose-response meta-analysis of the relationship between BMI and GBC risk (p=0.769 by Egger's test) GBC, gallbladder cancer.

No evidence for publication bias was indicated by Egger's regression test in the literature on BMI and GBC risk in overweight group (p=0.398) and dose-response group (p=0.769) (Figure 6a & Figure 7). For BMI and GBC risk in the obesity group, the Egger's test showed the possibility of publication bias for the analysis (p=0.008)(Figure 6b). because of this, we undertook the “trim and fill” analysis, and data was unchanged, suggesting that the effect of publication bias could be negligible.

## DISCUSSION

In our meta-analysis, we observed a statistically significant 10% greater risk of GBC in overweight individuals and a 58% greater GBC risk in obese individuals when compared with their normal-weight peers. Dose-response meta-analysis showed that each 1 kg/m^2^ increase was associated with 4% greater risk of GBC for overall. When adjusted for sex, at the point of BMI=25 kg/m^2^, the RRs for women and men were 1.13 and 0.98 respectively. The corresponding RRs at the point of BMI=30 kg/m^2^ were 1.56 vs. 1.24.

A meta-analysis by Larsson *et al.* [32] and Tan *et al.* [33] examined the association between BMI and risk of gallbladder cancer. They both reported a similar summary for overweight of obese individuals respectively. However, they did not examine the possibility of dose-response relationships between BMI and risk of gallbladder cancer. The World Cancer Research Fund (WCRF) recently updated its review [34] on gallbladder cancer risk factors. Our meta-analysis differs from the review of WCRF in some important aspects. Firstly, we evaluated the risk of gallbladder cancer in overweight individuals and obesity individuals, which indicates the obesity but not the overweight demonstrates a significant association with the risk of gallbladder cancer. This point is not mentioned in the WCRF report. Secondly, the WCRF report included only 8 studies, including 2 studies refereeing relationship of GBC mortality and BMI, which adds heterogeneity to the overall analysis for GBC incidence and BMI. Thirdly, we compared the risk of gallbladder cancer in different data resource in detail, including the study design, duration of follow-up, assessment methods of weight and adjustment factor, which make the result more solid.

Our meta-analysis has several potential limitations that may affect the interpretation of the results. First, overweight and obesity are typically associated with unhealthy diets but very few studies adjusted for intake of food; thus, these subgroup analyses are difficult to interpret. Besides, gallstones and increased use of laparoscopic cholecystectomy i.e. increases the risk of gallbladder cancer [15]. Meanwhile, obesity tends to be accompanied with diabetes, which is also associated with increased GBC risk [35]. However, most studies did not adjust for these risk factors. This could have led to an overestimation of the true association between obesity and risk of GBC. Second, weight and height data of several studies in this meta-analysis relied on self-reported and hospital discharges, which may attenuate the relative risk estimates. However, the RRs for BMI ascertained by measurement were similar to those by self-reported and hospital discharges. Finally, in our meta-analysis including only published studies, it is inevitable that an observed effect might suffer from publication bias because studies with null results tend not to be published. Interestingly, the “trim and fill” analysis showed that publication bias did not appreciably affect our results.

In summary, our meta-analysis indicates that the association of obesity and GBC is stronger in woman than in man. Furthermore, overweight is only associated with GBC in woman. A even stricter weight control might be necessary for woman to prevent GBC.

## MATERIALS AND METHODS

### Search strategy

We systematically searched PUBMED and EMBASE databases to April 17, 2016 for studies on the relationship between BMI and GBC risk. Our core search consisted of terms related to ‘gallbladder cancer, gallbladder neoplasm’ combined with ‘body mass index, BMI, overweight, or obesity’ to identify eligible studies. No language limits were set. In addition, all references listed in the retrieved articles and the reference lists of published meta-analysis [12, 32] were also scanned to further identify possible relevant publications.

### Study selection

Studies were eligible for inclusion in the meta-analysis if they satisfied the following criteria: (a) cohort or case–control studies study in which GBC incidence was taken as outcome; (b) having clear description of normal weight, overweight and obesity defined by BMI; (c) the studies reporting risk estimates with the corresponding 95% confidence intervals (95%CIs) or sufficient information to calculate them. When multiple studies had the same or overlapping study populations, only the studies contained the largest sample size or mostly completed were finally included.

### Data extraction

One investigator (ZML) extracted data, which was checked by another (LSW) and any disagreements were resolved by consensus. The following information was extracted from each of the eligible publications: first author's name, publication year, study location, ethnicity of population, follow-up years, age, measure method of BMI, sample size of gender, BMI categories and risk estimate for each BMI category, and covariates adjusted for multivariable analysis. We assumed that rate ratio and hazard ratio were all valid estimates of the relative risks (RRs), and we, therefore, reported all results as RR for simplicity. We extracted the relative risks with their 95% CIs that reflected the greatest degree of adjustment for potential confounders.

The midpoint of the upper and lower boundaries of each category was assigned as the mean BMI to each corresponding RRs of every study. If the upper boundary for the highest category (such as ≥30) and the lower boundary for the lowest category(such as<18.5) were not provided in the articles, we assumed that the boundary had the same amplitude as the adjacent category [36]. The method described by D.Aune was used to estimate the distribution of cases or person-years in studies that did not report these but reported the total number of cases and person-years [37].

### Statistical analysis

We used the WHO [38] classification to compare risk estimates for underweight(<18.5 kg m^−2^), overweight (25.0 to 29.9 kg m^−2^) and obesity (≥30.0 kg m^−2^) with normal weight (18.5 to 24.9 kg m^−2^). Where non-standard categories of BMI were used, we choose the category that was most similar to those defined by the WHO. The relative risks and corresponding standard errors from individual studies were logarithmically transformed to stabilize variances and normalize the distributions. Data were analyzed, and the results were reported, using a random-effects model [39]. To investigate the effect of potential confounders, subgroup analyses were conducted by the available characteristics of studies and participants.

For dose-response analysis, a two-stage random-effects dose-response meta-analysis [40] was performed to compute the trend from the correlated log RR estimates across levels of BMI, taking into account the between-study heterogeneity. In the first stage, a restricted cubic spline model with three knots at percentiles 10, 50 and 90% of the distribution was estimated using generalized least-square regression taking into account the correlation within each set of published RRs. Then, the GLST command with the generalized least-squares regression, which required the cases, person-years and mean level of BMI in each category, as well as the BMI level-specific RRs with variance estimated for at least three quantitative categories [41] of each article was used to carry out the dose-response meta-analysis. A p value for nonlinearity was calculated by testing the null hypothesis that the coefficient of the second spline was equal to zero [42].

We assessed heterogeneity between studies with the I^2^ statistic [43] as a measure of the proportion of total variation in estimates that is due to heterogeneity, where I^2^ values of 25%, 50%, and 75% correspond to cut-off points for low, moderate, and high degrees of heterogeneity.

Using meta-regression analysis, we further investigated whether the association between BMI and GBC was modified by study-specific factors, including study design and sex. We conducted a sensitivity analysis, in which one study at a time was removed and the rest analyzed to assess whether the results were markedly affected by a single study. Evidence of publication bias was assessed by visual inspection of funnel plots using Egger's regression test [44].

All statistical analyses were performed by Stata 12.0 (Stata Corporation, College Station, TX) and P values of two-sided less than 0.05 were considered statistically significant.

### Study quality score

We assessed the methodological “quality” of included studies based on the Newcastle-Ottawa Scale [45] for quality of case–control studies and cohort studies in meta-analysis; for this assessment, we used the Newcastle-Ottawa Scale star system (range, 0 to 9 stars). In the current study, we considered a study awarded seven or more stars as a high-quality study, because standard validated criteria for important end points have not been established.
